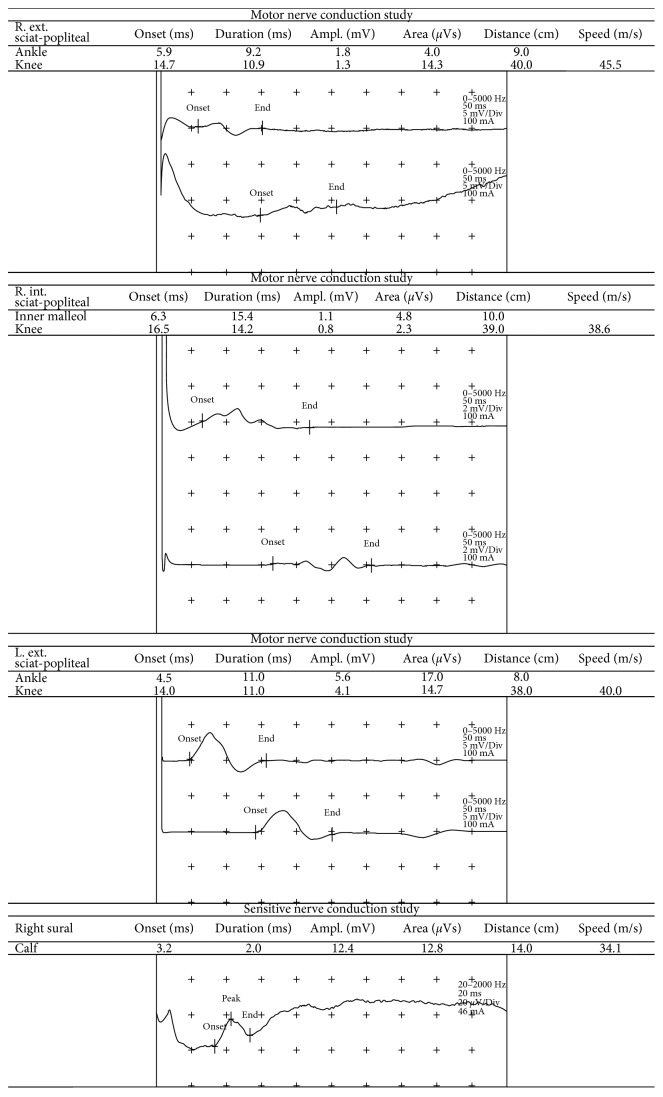# Safe Administration of Ipilimumab, Pembrolizumab, and Nivolumab in a Patient with Metastatic Melanoma, Psoriasis, and a Previous Guillain–Barré Syndrome

**DOI:** 10.1155/2018/2783917

**Published:** 2018-03-08

**Authors:** Alessio Cortellini, Alessandro Parisi, Maria Concetta Fargnoli, Katia Cannita, Azzurra Irelli, Giampiero Porzio, Claudio Martinazzo, Corrado Ficorella

**Affiliations:** ^1^Medical Oncology Unit, St. Salvatore Hospital, L'Aquila, Italy; ^2^Department of Biotechnological and Applied Clinical Sciences, University of L'Aquila, L'Aquila, Italy; ^3^Oncological Dermatology Unit, St. Salvatore Hospital, L'Aquila, Italy; ^4^Department of Neurophysiopathology, St. Salvatore Hospital, L'Aquila, Italy

## Abstract

**Background:**

Patients with autoimmune diseases were not evaluated in clinical trials with immune checkpoint inhibitors (ICIs), since a history of immune disorders, such as Guillain–Barré syndrome (GBS) and psoriasis, is one of the major risk factors for the development of immune-related adverse events (irAEs). This risk cannot be defined; therefore, physicians are called to manage these patients in clinical practice.

**Case Report:**

We report the case of a 62-year-old male patient affected by metastatic melanoma, with a history of GBS and psoriasis, and treated with sequential ipilimumab, pembrolizumab, and nivolumab, without significant toxicities.

**Conclusion:**

This case report supports that although a history of immune disorders is one of the major risk factors for development of irAEs, in some patients, it could be possible to safely administer sequential treatments with ICIs. A proper decision should be made, considering therapeutic options, disease-related risks, and those related to a recurrence of preexisting autoimmune disorders.

## 1. Introduction

Before starting a treatment with immune checkpoint inhibitors (ICIs), oncologists must identify potential risk factors, such as previous or concomitant dysimmune disorders, that could favour the development of immune-related adverse events (irAEs). Unfortunately, patients with a history of autoimmune diseases were not included in clinical trials; however, after careful baseline assessment, they are more frequent than expected in common clinical practice. In this case, proper management, early diagnosis, and careful pre- and post-treatment monitoring of irAEs are required [1]. IrAEs are reported more frequently with anti-CTLA4 (cytotoxic T-lymphocyte-associated antigen 4) monotherapy rather than with anti-PD-1/PD-L1 (programmed death-1/programmed death-ligand 1) [2]. Immune-mediated polyneuropathies are more frequently related to ipilimumab than to nivolumab or pembrolizumab; they are rare, occurring approximately in 1% of patients and up to 4.5% when referring to all neurological toxicities [2–5]. Guillain–Barré syndrome (GBS) is an acute polyradiculoneuropathy with variable clinical presentation. The pathogenesis of GBS is unclear, but it is well known that it is caused by cellular and humoral immune self-response against peripheral nerves. GBS could be considered as an exceptional irAE with only five cases reported [6–10]. Numerous triggering events have been described, such as infections; GBS can lead to death as a result of complications (infections, thromboembolic events, respiratory failure, and cardiac arrhythmias) in about 5% of cases [11]. Skin disorders are the most frequent toxicity of ICIs: overall incidence of dermatological irAEs appeared to be similar with anti-CTLA4 and anti-PD-1/PD-L1. Considering any grade, they occur from 10% to 60% (in combination therapy) of patients [3–5, 12–15]. Most cutaneous irAEs are mild, reversible, and easily manageable following guidelines; they are often T-cell-mediated even if the pathophysiology is still unknown. Psoriasis is a multifactorial immune-mediated chronic cutaneous disease, characterized by a wide range of clinical manifestations from mild to severe forms. Worsening and recurrence of psoriasis have been reported during the use of ICIs, with both anti-CTLA4 and anti-PD-1, such as nivolumab [16–20]. Recently, a case series of advanced melanoma patients treated with anti-PD-1 therapy and with preexisting autoimmune disorders has included 2 patients with a history of GBS (none of them experienced a worsening/flare) and 6 patients with a history of psoriasis (3 of them experienced cutaneous irAEs) [21]. We report the case of a 62-year-old male patient, with metastatic melanoma and a history of GBS and psoriasis. The patient was treated with sequential ipilimumab, pembrolizumab, and nivolumab, without significant toxicities or worsening of the preexisting autoimmune disorders. The patient was treated in clinical practice with “in-label” drugs in Italy and provided written informed consent to the proposed treatment; procedures followed in reporting the case are in accordance with the ethical standard of the local responsible committee on human experimentation.

## 2. Case Presentation

We report the case of a male patient, a smoker, with a history of chronic obstructive lung disease, atrial fibrillation, hypertension, obesity, chronic plaque psoriasis, and Guillain–Barré syndrome (GBS). The diagnosis of GBS dated back to 2002; during a community-acquired pneumonia, a molecular mimetism between bacterial antigens and gangliosides of the nerves' myelin sheath led to the development of a severe and rapidly progressive muscle weakness with areflexia, till tetraplegia. Electromyography (EMG) confirmed acute, axonal polyneuropathy, with reduced sensory action potential, supporting the diagnosis of the “acute motor and sensory axonal neuropathy” (AMSAN) type of GBS. The patient was hospitalized and successfully treated with intravenous immunoglobulins; he then underwent upper left lobectomy of the lung, in order to excise a bronchiectasis, which was acting as a reservoir of bacteria. Besides a residual neurological injury to his legs, no recurrences were later observed. The patient also reported a history of moderate-to-severe plaque psoriasis, previously treated with cyclosporine A, which was stopped in 2013.

In February 2015, he underwent surgical resection of cutaneous melanoma of the left gluteus, with the following histopathological features: nodular melanoma, ulcerated, Breslow thickness 9 mm, poorly pigmented, 12 mitoses/mm^2^, Clark's level IV, without regression and intra/peritumoral lymphocytic infiltrate pT4b [22]. Wide surgical excision and sentinel lymph node biopsy were negative for metastatic involvement (pathological stage IIC). In July 2015, positron-emission tomography (PET scan) showed pathological enhancement of the retroperitoneal and left inguinal lymph nodes (standardized uptake values from 2.6 to 22.5); therefore, in September 2015, he underwent wide lymphadenectomy, resulting in 23 out of 42 metastatic lymph nodes; PET scan in December 2015 still demonstrated residual disease in the retroperitoneal lymph nodes. He came to our attention at the age of 62, in quite good clinical conditions, with eastern cooperative oncology group performance status (ECOG-PS) 1. The *BRAF* mutational analysis (V600) was negative (cobas® z 480 analyzer). The high tumor burden, with LDH more than 2 ULN, and the absence of an actionable *BRAF* mutation led us to choose a first-line treatment with immune checkpoint inhibitors (ICIs). At that time, the only “in-label” drug in Italy for first-line treatment of *BRAF* wild-type advanced melanoma patients was ipilimumab (Yervoy®; Bristol-Myers Squibb Pharma EEIG, Uxbridge, United Kingdom). Before starting ipilimumab, we made a careful multidisciplinary assessment with dermatological and neurological evaluation. On dermatological evaluation, the patient presented psoriatic plaques on the trunk and extremities; a baseline EMG was performed that showed the residual functional loss, mainly to the legs (Figure 1).

From December 11, 2015, to February 19, 2016, 4 induction doses of ipilimumab (3 mg/kg every three weeks) were administered without significant toxicities, except development of cutaneous facial vitiligo on the face; psoriatic plaques remained unchanged, and the patient did not develop new neurological symptoms. A CT scan in March 2016 showed an immune-related response of disease (dimensional reduction and intralesional necrosis) and also detected a pulmonary embolism, treated with low molecular weight heparin. In April 2016, the CT scan showed resolution of pulmonary embolism, and neurological evaluation showed no changes, just like the control EMG that was performed.

In June 2016, the CT scan showed progressive disease to the lymph nodes; therefore, the patient underwent a second-line therapy with pembrolizumab (2 mg/kg every three weeks) (Keytruda®; Merck Sharp & Dohme Limited, Hoddesdon, United Kingdom), with only one dose administered, on July 11. After that, he decided to continue the treatment at an outpatient cancer care center closer to his home. From October to November 2016, he received 3 doses of nivolumab (3 mg/kg every two weeks) (Opdivo®; Bristol-Myers Squibb Pharma EEIG, Uxbridge, United Kingdom), without developing significant toxicities, but he died in December 2016 due to the progression of the disease.

## 3. Discussion

In clinical practice, every patient should be carefully interrogated about the personal and family history of immune disorders because it is one of the few acknowledged risk factors for development of irAEs [1]; following that diagnosis, it is impossible to quantify the risk of worsening/recurrence of the preexisting autoimmune disease. Treatment decision should be made, properly weighing the expected clinical outcome and safety profile in each patient. This kind of patients must be carefully monitored during a treatment with ICIs, in close collaboration with organ-specific consultants, in order to diagnose as soon as possible a potential irAE. Our patient had a history of GBS, a serious and life-threatening disease, and at the same time a moderate psoriasis. The melanoma-related risk of developing symptoms and of death was higher than that of developing an irAE; therefore, the absence of *BRAF* actionable mutations forced us to start a first-line treatment with ipilimumab, followed by pembrolizumab and nivolumab after disease progression. All the ICIs were well tolerated, without significant toxicities.

## 4. Conclusion

This case report supports the idea that, in some patients, it could be possible to safely administer sequential treatments with ICIs, although a history of preexisting immune disorders is one of the major risk factors for the development of irAEs. The decision-making process should include a proper balance between the safety profile and expectations; alternatives should be discussed with patients and families, whose compliance is fundamental to reach a good clinical outcome.

## Figures and Tables

**Figure 1:**